# The intestinal microbiota contributes to the growth and physiological state of muscle tissue in piglets

**DOI:** 10.1038/s41598-021-90881-5

**Published:** 2021-05-27

**Authors:** Renli Qi, Jing Sun, Xiaoyu Qiu, Yong Zhang, Jing Wang, Qi Wang, Jinxiu Huang, Liangpeng Ge, Zuohua Liu

**Affiliations:** 1 Chongqing Academy of Animal Science, Rongchang, Chongqing, 402460 China; 2 Key Laboratory of Pig Industry Sciences, Ministry of Agriculture and Rural Areas, Rongchang, Chongqing, 402460 China; 3 Chongqing Key Laboratory of Pig Industry Sciences, Rongchang, Chongqing, 402460 China; 4 Southwest University of Science and Technology, Mianyang, 621010 Sichuan China

**Keywords:** Biogeochemistry, Animal physiology, Microbiology

## Abstract

Although the importance of the intestinal microbiota in host growth and health is well known, the relationship between microbiota colonization and muscle development is unclear. In this study, the direct causal effects of the colonization of gut microorganisms on the muscle tissue of piglets were investigated. The body weight and lean mass of germ-free (GF) piglets were approximately 40% lower than those of normal piglets. The deletion of the intestinal microbiota led to weakened muscle function and a reduction in myogenic regulatory proteins, such as MyoG and MyoD, in GF piglets. In addition, the blinded IGF1/AKT/mTOR pathway in GF piglets caused muscle atrophy and autophagy, which were characterized by the high expression of Murf-1 and KLF15. Gut microbiota introduced to GF piglets via fecal microbiota transplantation not only colonized the gut but also partially restored muscle growth and development. Furthermore, the proportion of slow-twitch muscle fibers was lower in the muscle of GF piglets, which was caused by the reduced short-chain fatty acid content in the circulation and impaired mitochondrial function in muscle. Collectively, these findings suggest that the growth, development and function of skeletal muscle in animals are mediated by the intestinal microbiota.

## Introduction

An immense number of microorganisms, collectively known as the microbiota, colonize the intestines of humans and animals after birth. This community consists of at least 10^13^ individuals, is predominated by anaerobic bacteria, and includes 500–1000 species^1,2^. The abundance and diversity of the gut microbiota can be highly related to the host’s physiology, and these microorganisms may perform functions that the hosts have not had to evolve, including the ability to process otherwise indigestible components of the host’s diet, such as plant polysaccharides^3,4^. The gut microbiota also has critical roles in the maintenance of host health by promoting host cell differentiation, protecting the host from the colonization of pathogens, and stimulating/modulating the immune system^5–8^. A large number of microbial metabolites [short-chain fatty acids (SCFAs), organic salts, amines, alkaloids] are produced in the digestive tract. Bacterial metabolites can enter the circulatory system and participate in a variety of complex physiological and biochemical reactions at different sites, including interference with nerve signal transduction and hormone secretion^9–11^.

The interaction between the gut microbiota and organs has been a focus of recent scientific research. There are many studies investigating how the gut microbiota affects the gut, immunity, liver and brain^4,6,7,11,12^. However, few studies have reported the effects of gut microbiota on skeletal muscle. In mammals, skeletal muscles have the main function of contracting to facilitate the movement of the body, thus leading to energy expenditure. Therefore, existence of a gut-muscle communication pathway has been suggested^13^. A number of reports have implied that intestinal microbiota control the growth and function of muscle tissue in humans and animals. In a recent report, germ-free (GF) mice exhibited muscle atrophy, decreased expression of insulin-like growth factor (IGF) and reduced expression of genes related to skeletal muscle growth and mitochondrial function^14^. An imbalance or a reduction in gut microbiota diversity could also lead to muscular dysplasia and dysfunction in humans and rodents^15,16^. Pigs serve as a major meat source in many countries and are also an ideal animal model for physiological and pathological studies based on their similarity to humans in terms of digestive and metabolic characteristics^17,18^. However, investigations of the role of the intestinal microbiota in the muscle development of pigs are limited to date.

GF pigs are an experimental sterile large animal model with a natural advantage: the absence of gut microflora^19,20^. In previous studies, GF pigs were used to reveal the influence of microbiota on the formation and maturation of the immune system mainly^21,22^. The principal aim of this study was to reveal the direct causal effects of intestinal microbiota colonization on pig muscle tissue. Early growth and development of skeletal muscle in piglets with or without gut microbiota were assessed. GF piglets were employed in comparison with piglets with gut bacteria. In addition, a group of GF piglets were provided with intestinal microbiota by the fecal microbiota transplantation (FMT) method after birth to evaluate the compensatory effect of microbiota colonization on muscle growth. By comparing the different pigs, we demonstrated that the growth, development and function of muscles are mediated by the stable and diverse intestinal microbiota to a certain extent.

## Results

### Physiological and biochemical indicators in piglets

**Figure 1:**
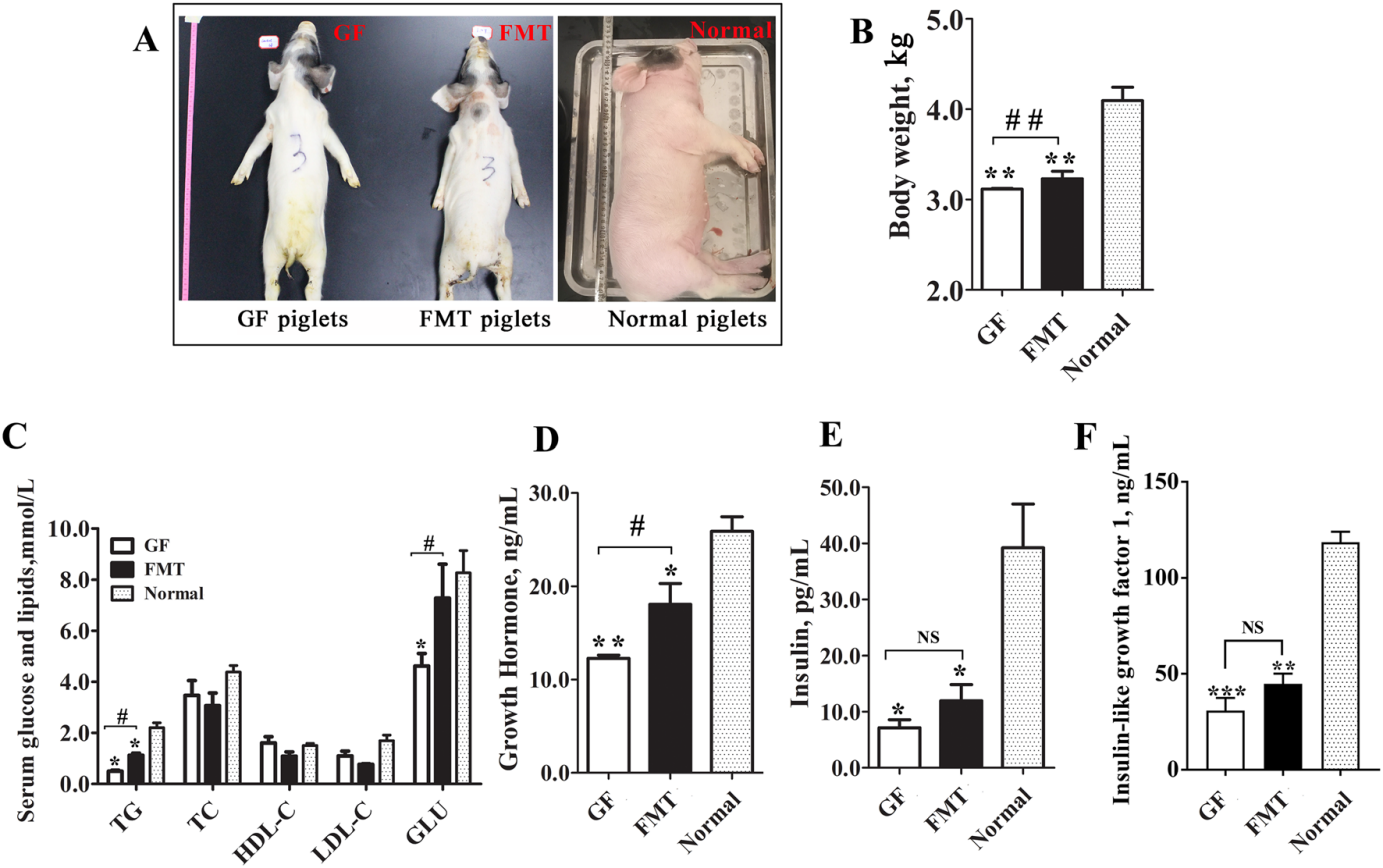
Comparison of physiological biochemical indicators of different piglets. (**A**) Body size. (**B**) Body weight. (**C**) Blood biochemical parameters. (**D**) Concentration of growth hormones in the blood of piglets. (**E**) Concentration of insulin in the blood of piglets. (**F**) Concentration of insulin-like growth factor 1 (IGF1) in the blood of piglets. N = 3, the data are presented as the means ± s.e.m. *P < 0.05, **P < 0.01 compared to the normal piglets (control). ^#^*P* < 0.05, ^##^*P* < 0.01 between the GF piglets and FMT piglets. *NS* not significant, *GF piglets* germ-free piglets, *FMT piglets* GF piglets with fecal microbiota transplantation, *TC* total cholesterol, *TG* triglycerides, *HDL-C* high-density lipoprotein cholesterol, *LDL-C* low-density lipoprotein cholesterol, *GLU* glucose.

The GF piglets exhibited lower growth rates than the normal piglets (as controls) with a gut microbiota but did not show other obvious metabolic diseases during the experimental period (birth to 25 days old). The GF piglets treated with FMT exhibited partially restored growth in the later days. The normal piglets exhibited good growth statuses and health physiological indicators throughout the study. However, the GF and FMT piglets were smaller and thinner than the control piglets of the same age. A comparison of the young piglets in the three groups indicated that the average body weight (BW) of the normal and FMT piglets was ~ 1.40-fold (*P* = 0.0010) and ~ 1.16-fold (*P* = 0.0019) higher than that of GF piglets (Fig. 1A,B). In addition, the GF piglets exhibited the lowest serum levels of triglycerides (TG), glucose, and hormones [growth hormone, insulin, insulin-like growth factor 1 (IGF1)] among the three groups (Fig. 1C–F). Although FMT did not completely repair the growth status of the GF piglets, the FMT piglets showed better body conditions and physiological traits than the GF piglets. The colonization of the gut microbiota in the FMT piglets resulted in elevated development indexes. In addition to BW, the blood concentrations of TG, glucose, and growth hormone in the FMT piglets were also significantly higher than those of GF piglets (*P* < 0.05, Fig. 1C–F). These results suggest that the intestinal microbiota is essential for the normal growth and development of piglets.

### Intestinal microbiota and metabolites

**Figure 2:**
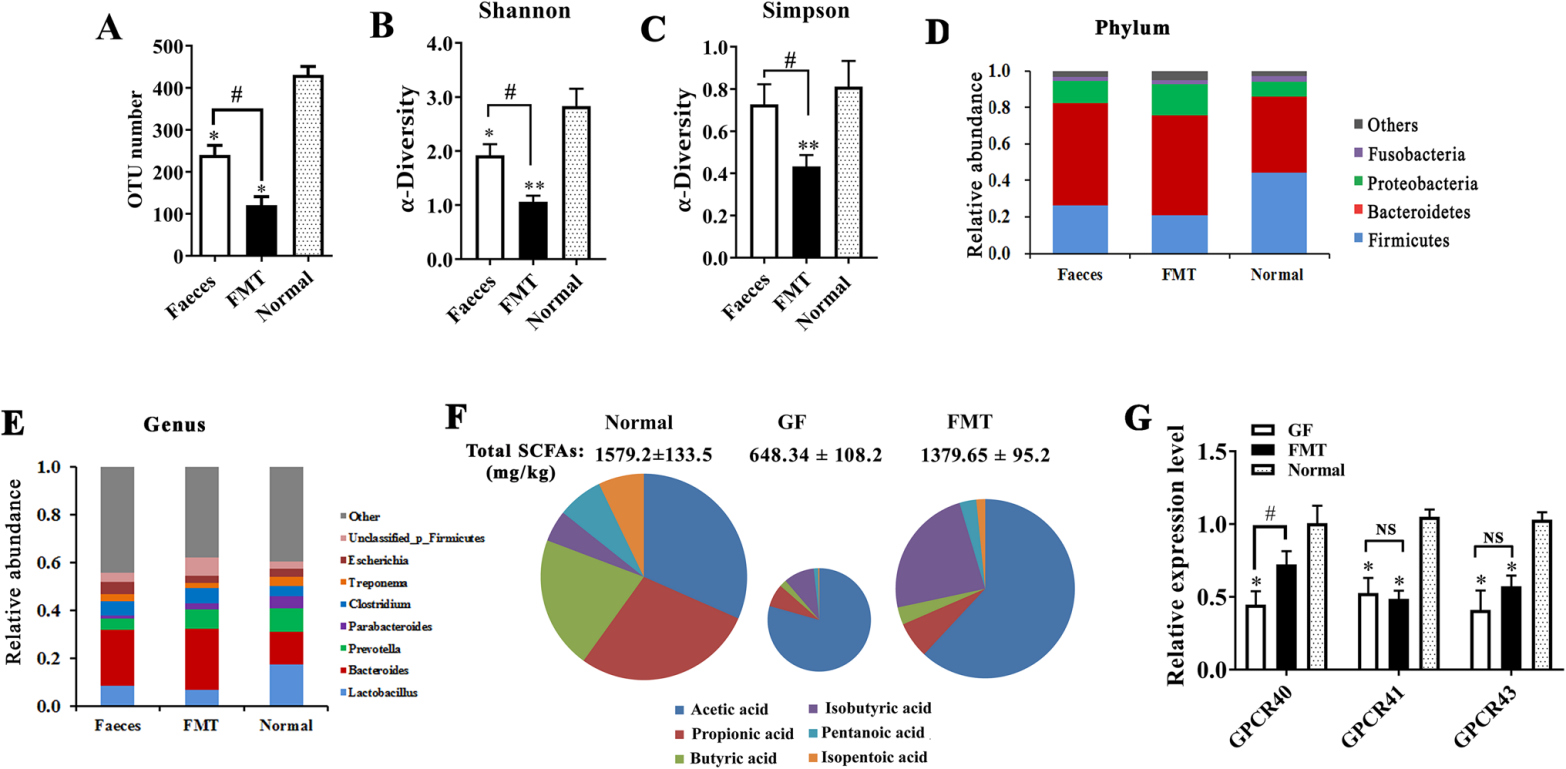
Colonization of the intestinal microbiota in GF piglets by FMT. (**A**) Operational taxonomic unit (OTU) number of gut bacteria in the colon digesta of piglets. (**B**) and (**C**) α-Diversity of intestinal bacteria. (**D**) and (**E**) Bacterial composition of the different communities at the phylum level and genus level. (**F**) The content of short-chain fatty acids (SCFAs) in the colonic content of the piglets. (**G**) Expression levels of G-protein-coupled receptors (GPCRs). mRNA expression was determined by qPCR in the colonic wall. N = 3, the data are presented as the means ± s.e.m. *P < 0.05, **P < 0.01 compared to the normal piglets (control). ^#^*P* < 0.05, ^##^*P* < 0.01 between the GF piglets and FMT piglets. *SCFAs* short-chain fatty acids, *NS* not significant, *GF piglets* germ-free piglets, *FMT piglets* GF piglets with fecal microbiota transplantation.

To help GF piglets gain an intestinal microbiota, we prepared and transferred bacteria from a healthy swine fecal sample to three GF piglets by oral gavage on the 3rd to 7th day after birth. 16S rRNA sequencing showed that more than 110 operational taxonomic units (OTUs) were detected at the genus level in the colonic digesta of the 25-day-old FMT piglets. However, the OTU number in the FMT piglets was still less than that in normal piglets of the same age (*P* < 0.05, Fig. 2A). Clearly, the diversity and richness of the intestinal microbiota in FMT piglets were also significantly lower than those of normal piglets (Fig. 2B,C). In addition, the bacterial microbiome structures of the FMT piglets and normal piglets were not exactly the same. The abundance of the *Firmicutes* phylum was significantly reduced, while the *Bacteroidetes* and *Proteobacteria* phyla increased in the colon digesta of FMT piglets compared with normal piglets (Fig. 2D). Several dominant bacterial genera showed changes in abundance in FMT piglets compared with normal piglets. The relative abundance of *Lactobacillus* was lower and *Bacteroides* was higher in FMT piglets than in normal piglets (Fig. 2E).

SCFAs are also referred to as volatile fatty acids and are mainly composed of acetate, propionate, and butyrate. SCFAs are used as potential energy sources, represent the major fuel for enterocytes and play key roles in regulating host metabolism, the immune system, and cell proliferation^23–25^. Our data showed that the total SCFAs content in the colon of GF piglets was significantly lower than that in normal piglets (Fig. 2F). Microbiota colonization caused a significant increase in the total SCFA content in the FMT piglets (~ twofold of GF piglets, *P* < 0.01), and the amount of total SCFAs in the FMT piglets was close to that in normal piglets. However, we also noticed that there was a clear difference in the SCFA composition among the three groups. Acetic acid was approximately 37% of the total SCFAs content in the colonic digesta of the normal piglets; however, the proportion of acetic acid exceeded almost two-thirds of the total SCFAs content in the GF piglets and FMT piglets. The butyric acid contents in the GF piglets and FMT piglets were only less than one-tenth of those of the normal piglets. We also determined the expression levels of GPCR40, 41, and 43, major receptor proteins for SCFAs in the cellular membrane^26,27^. The data showed that the expression levels of these genes in the distal gastrointestinal tract of GF piglets were lower than those in normal piglets and FMT piglets (Fig. 2G). All of these results indicate that FMT successfully replanted microbes in the gut of GF piglets; however, the richness and diversity of the gut microbiota in FMT piglets were not sufficient.

### Development and function of muscle tissue

**Figure 3:**
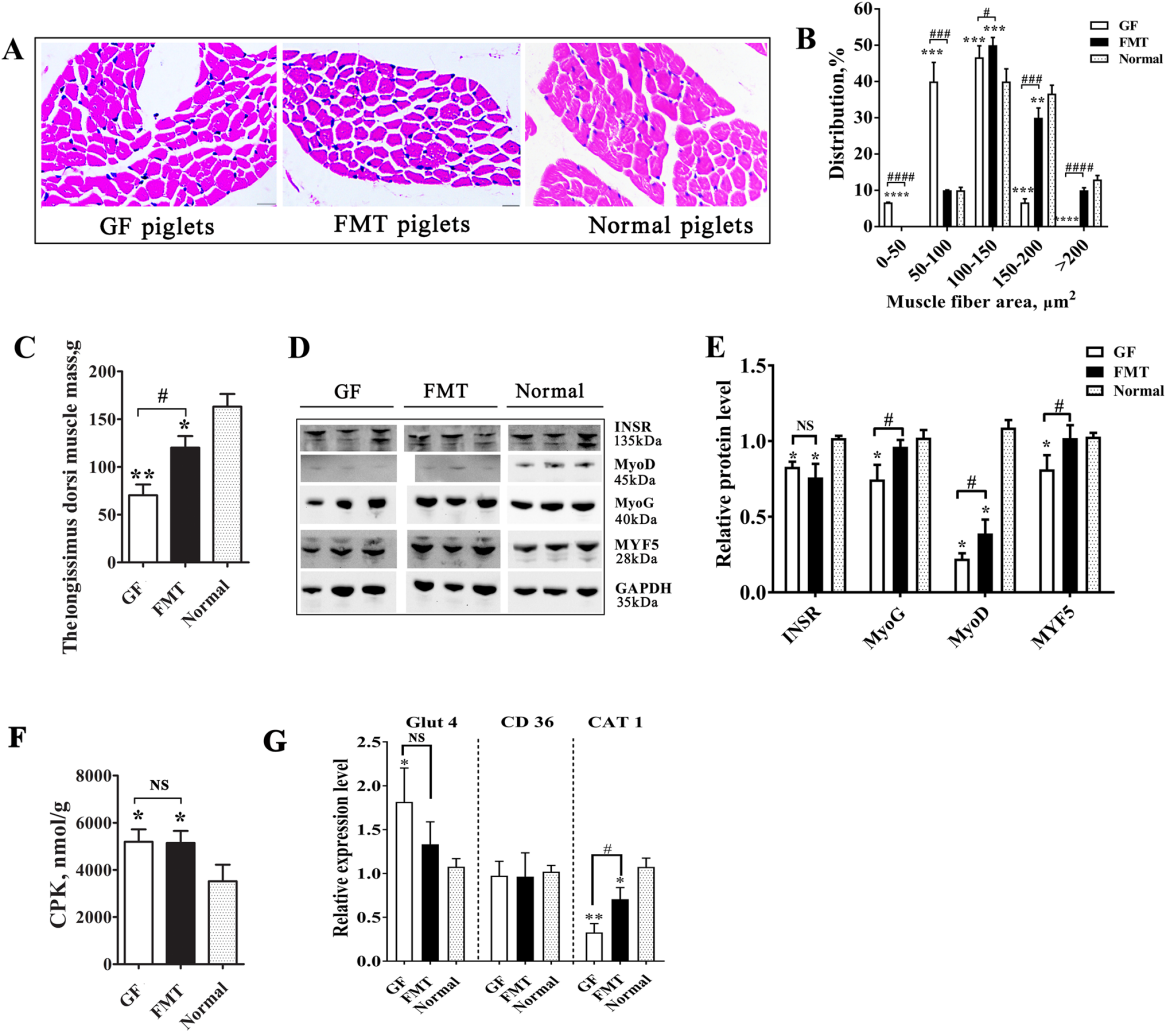
Comparison of muscle growth in different piglets. (**A**) Sections of the longissimus dorsi muscle with hematoxylin–eosin (HE) staining. (**B**) Sectional area of muscle fiber. (**C**) Weight of the left longissimus dorsi muscle mass. (**D**) Protein levels of key myogenic factors and insulin receptor (INSR) as detected by Western blotting. The full-length blots are presented in Supplementary Fig. 1. (**E**) Relative quantitative analysis of the protein levels. (**F**) Enzyme activity of creatine phosphokinase (CPK) in muscle tissue. (**G**) Expression levels of glucose transporter 4 (Glut4), fatty acid translocase (FAT/CD36) and cationic amino acid transporter 1 (CAT1). mRNA expression in the muscle tissue was determined by qPCR. N = 3, the data are presented as the means ± s.e.m. *P < 0.05, **P < 0.01, ***P < 0.001, and ****P < 0.0001 compared to the normal piglets (control). ^#^*P* < 0.05, ^###^*P* < 0.001, and ^####^*P* < 0.0001 between the GF piglets and the FMT piglets. *NS* not significant, *GF piglets* germ-free piglets, *FMT piglets* GF piglets with fecal microbiota transplantation.

The core aim of this study was to assess the direct influence of gut microbiota on the early growth and function of skeletal muscle in piglets (Fig. 3). The *longissimus dorsi* muscle mass was significantly smaller and the muscle fiber was thinner in the GF piglets than in the normal piglets (Fig. 3A–C). Western blot analysis indicated that the protein abundance of myogenic factor 5 (MYF5), myogenin (MyoG), and myogenic differentiation 1 (MyoD), three key myogenesis regulators, was lower in the muscles of GF piglets than in normal piglets (Fig. 3D,E). In particular, the protein level of MyoD in GF piglets was less than that in normal piglets (30%, *P* < 0.01). Although not completely comparable to the normal piglets, the FMT piglets also showed larger muscle mass and thicker muscle fibers (*P* < 0.05) than the GF piglets. The protein levels of myogenic factors in the FMT piglets increased accordingly (especially MYF5 and MyoG). The development and metabolism of skeletal muscle in mammals are closely controlled by insulin signals^28^. Insulin and IGFs in circulation bind to receptor proteins in different organs, which then activate downstream pathways^29^. We found that the insulin receptor (INSR) in the *longissimus dorsi* muscle also showed a reduction in protein levels in GF piglets (Fig. 3D).

Creatine phosphokinase (CPK) is an important enzyme involved in energy metabolism and is mainly found in skeletal muscle and the myocardium. CPK enzyme activity has been reported to be elevated in individuals with progressive muscular dystrophy, polymyositis or muscle damage^30^. Our data showed that the CPK level in GF piglet muscle was 1.47-fold that in normal piglets (*P* < 0.05, Fig. 3F), which was indicative of abnormal muscle function in GF piglets. We also investigated the nutrient uptake ability of muscle by detecting the expression levels of glucose transporters (glucose transporter 4, Glut4), fatty acid transporters (fatty acid translocase, FAT/CD36) and amino acid transporters (cationic amino acid transporter 1, CAT1)^31–33^. Higher expression of Glut4 and lower expression of CAT1 in the muscle were observed in GF piglets than in FMT and normal piglets (Fig. 3G), suggesting the strengthening of glucose uptake capacity and weakening of amino acid uptake capacity in piglets without gut microbes. Clearly, the insufficient intake of amino acids crippled the growth and anabolism of muscles. All these results indicated developmental retardation and dysfunction in pig muscle tissues caused by the absence of gut bacteria.

### Change in the gene expression profile in muscle tissue

**Figure 4:**
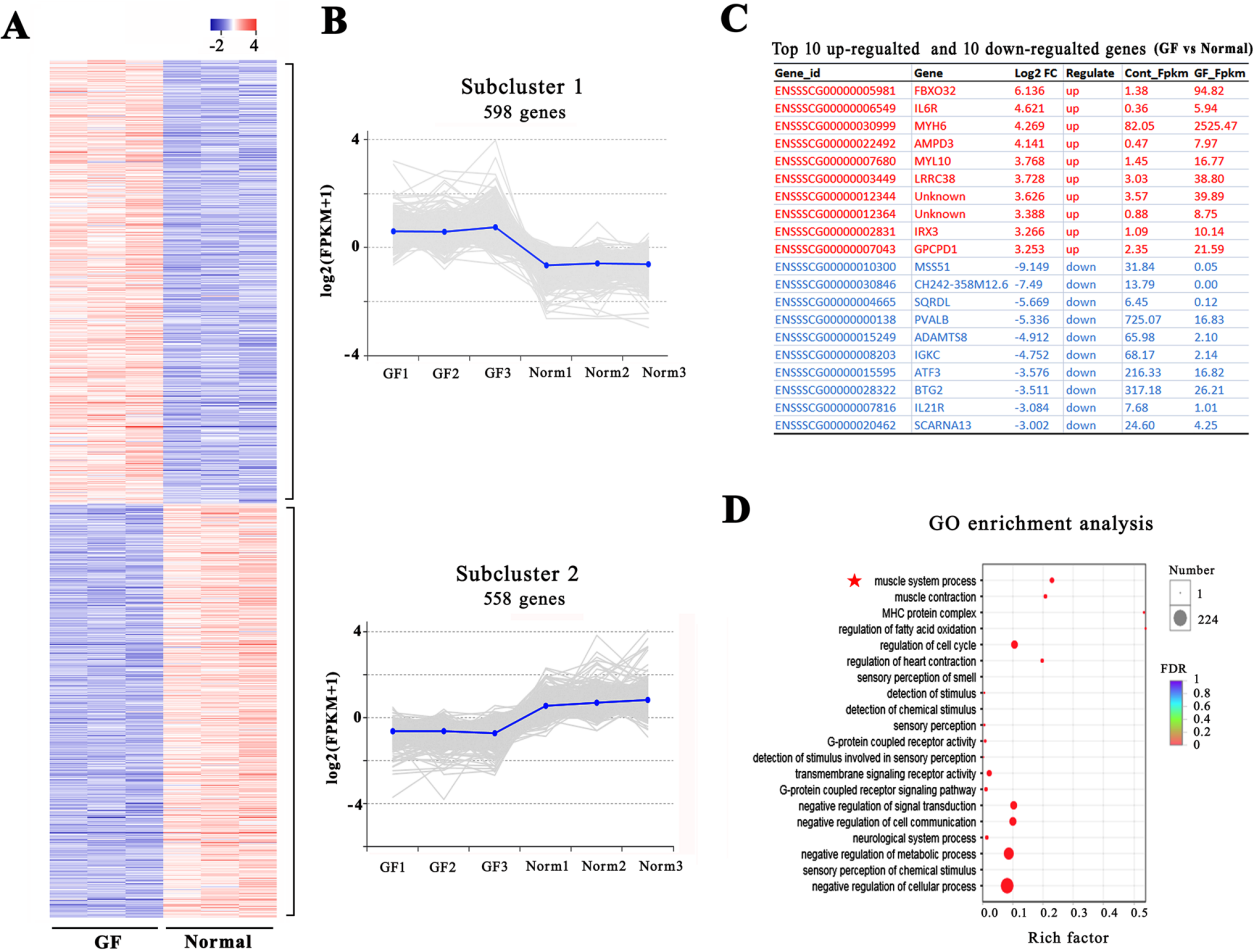
RNA-seq analysis of protein-coding gene expression in the muscle tissues of GF piglets and normal piglets. (**A**) Heatmap of 1156 significantly differentially expressed genes (DEGs). The red dots and the blue dots indicate up- or downregulated genes with fold changes > 2.0 and *P adjusted* < 0.05. (**B**) Abundance patterns of the genes segregated into two clusters after k-means analysis. (**C**) Top 10 upregulated genes and 10 downregulated genes in the muscle tissue of GF piglets vs. normal piglets. (**D**) DEGs enriched in the GO term muscle system process.

To further understand the differences in regulatory molecular networks, a high-throughput RNA-seq analysis was conducted to analyze the gene expression profiles in the skeletal muscles of GF and normal piglets. Regarding the expression profiles of protein-coding genes, 1039 mRNAs were detected only in the GF piglets, 1075 mRNAs were detected only in the normal piglets, and 13,667 mRNAs were present in both groups. A total of 1156 known protein-coding genes showed significant differential expression (*P adjusted* < 0.05 and fold-change > 2.0) in the muscles of GF piglets compared to normal piglets (Fig. 4A). K-means grouped all the differential mRNAs in two clusters based on their abundance patterns. Cluster 1 grouped 598 highly expressed genes, and cluster 2 grouped 558 lowly expressed genes in GF pigs (Fig. 4B). Figure 4C shows the top 10 upregulated genes and the top 10 downregulated genes in the GF piglets.

We further measured the classification and function of the differentially expressed genes (DEGs) by gene ontology (GO) enrichment analysis. We obtained more than 100 enriched GO terms with a false discovery rate (FDR) < 0.05. Figure 4D shows the top 20 significantly enriched GO terms. Many of them were closely related to the progressive regulation of muscle growth and development, including the MHC protein complex, muscle system process, fatty acid oxidation, and muscle contraction. In addition, the DEGs were also significantly enriched in the terms of cell cycle, metabolic process, G-protein signaling, and immune function.

**Figure 5:**
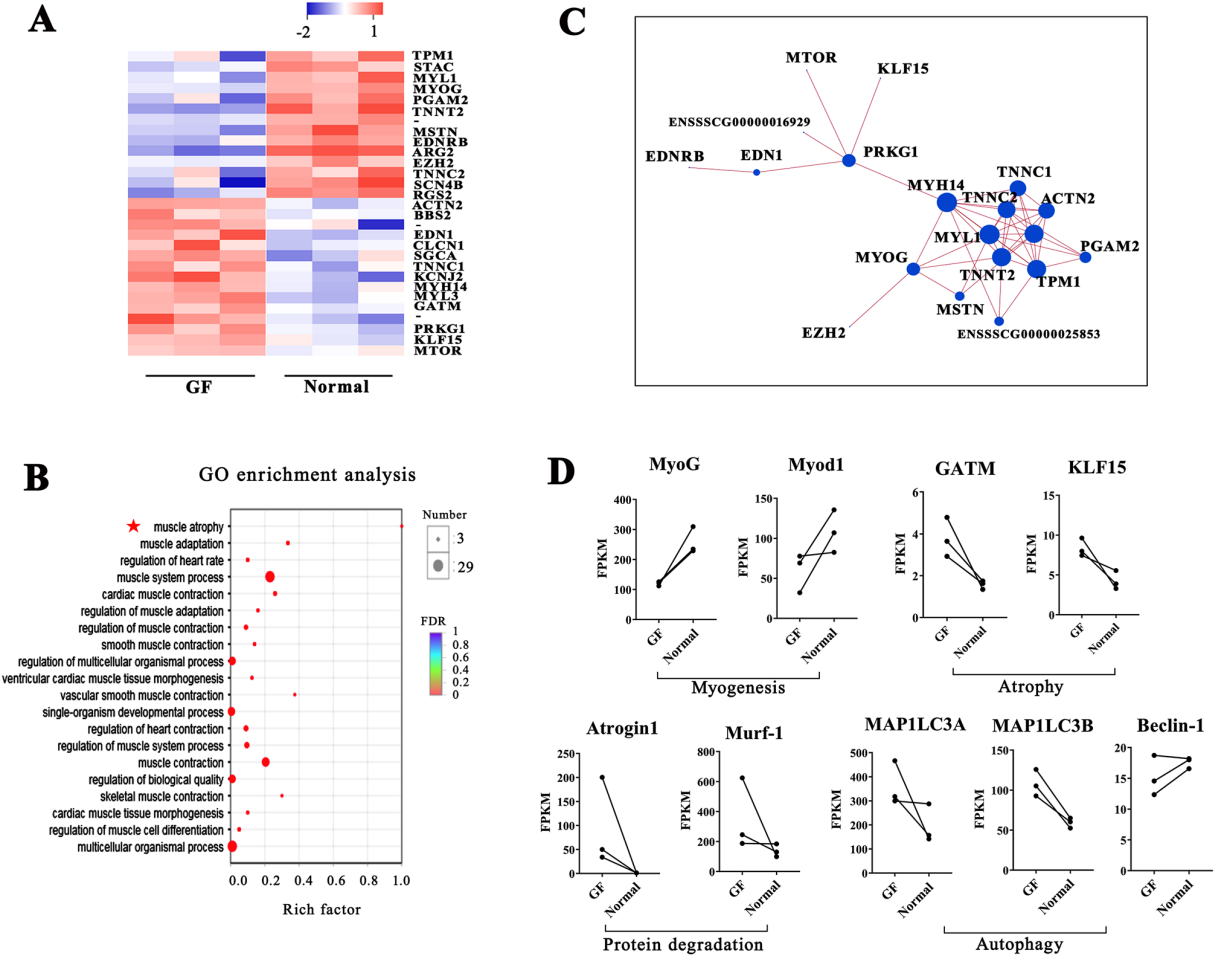
DEGs related to muscle atrophy, protein degradation and muscle autophagy. (**A**) Twenty-nine DEGs were enriched in the GO term "muscle system process". (**B**) Functional annotation for the twenty-nine muscle-related genes. (**C**) Protein interaction network constructed by twenty-nine genes involved in the muscle system. (**D**) Changes in key genes involved in myogenesis, muscle atrophy, protein degradation and muscle autophagy in GF piglets.

Twenty-nine DEGs were enriched in the GO term "muscle system process" and were, therefore, the core factors in the control network for muscle growth (Fig. 5A). We found that the changes in these genes caused muscle atrophy in GF piglets by the functional classification of DEGs (Fig. 5B). In addition, the significant changes in the protein-coding genes for myosin light chain (MYL) and myosin heavy chain (MYH) (such as MYL1 and MYH14) directly affected muscle fiber type and muscle metabolism. A protein–protein interaction network was generated with these 29 muscle-related genes (Fig. 5C). A decrease in MyoG and increases in KLF15^34^ and GATM^35^ promoted muscle atrophy in GF pigs (Fig. 5A,B). In addition, a number of functional genes related to the control of ubiquitin-mediated protein degradation (i.e., Atrogin1 and Murf1^36,37^) and muscle autophagy (i.e., MAPILC3A and MAPILC3B^38^) were changed in the GF pigs to different extents (Fig. 5D).

### Reduction and inactivation of IGF1/AKT/mTOR pathway

**Figure 6:**
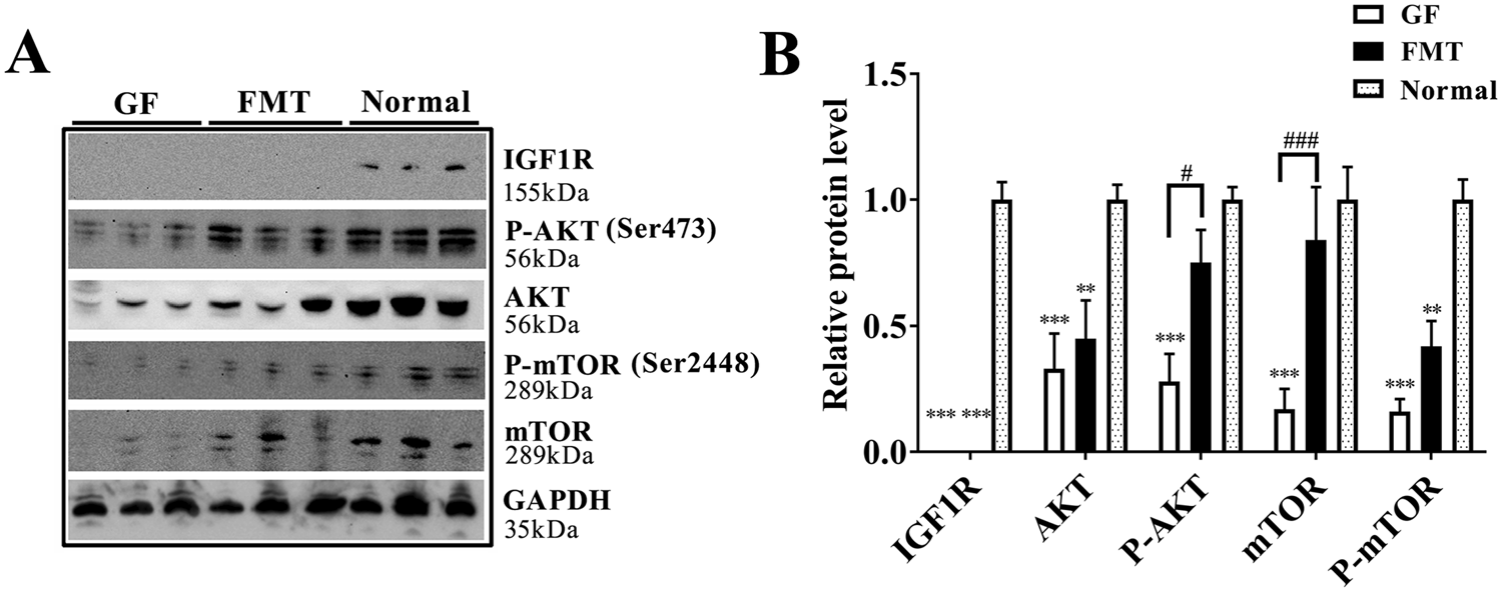
Changes of IGF1/AKT/mTOR pathway in muscle. (**A**) Protein levels of IGF1R, AKT, P-AKT, mTOR and P-mTOR were detected by Western blotting. The full-length blots are presented in Supplementary Fig. 1. (**B**) Relative quantitative analysis of the protein levels. The data are presented as the means ± s.e.m. N = 3, **P < 0.01, ***P < 0.001 compared to the normal piglets (control). ^#^*P* < 0.05 and ^#^*P* < 0.001 between the GF piglets and the FMT piglets. *GF piglets* germ-free piglets, *FMT piglets* GF piglets with fecal microbiota transplantation.

We next attempted to determine the underlying induction of muscle atrophy in GF piglets and thus analyzed the expression and activation of the IGF1/AKT/mTOR signaling pathway. This pathway serves to control and coordinate hypertrophic and atrophic messages in skeletal muscle and culminates in a delicate balance between muscle protein synthesis and proteolysis^39,40^. Insulin like growth factor 1 receptor (IGF1R), AKT serine/threonine kinase (AKT), and mammalian target of Rapamycin (mTOR), which are core members in the pathway, were reduced and significantly inactivated in the muscle tissue of GF piglets, and IGF1R was almost undetectable in GF and FMT piglets by the western blotting analysis (Fig. 6A,B). Obviously, this finding was consistent with the drastic decrease in IGF1 blood content in GF and FMT pigs (Fig. 1F). We have reason to believe that the continued lack of important hormones, such as IGF1, caused by the absence of gut microbiota could weaken protein synthesis and aggravate muscle atrophy by blinding the IGF1/AKT/mTOR pathway.

### Change of muscle fiber type

**Figure 7:**
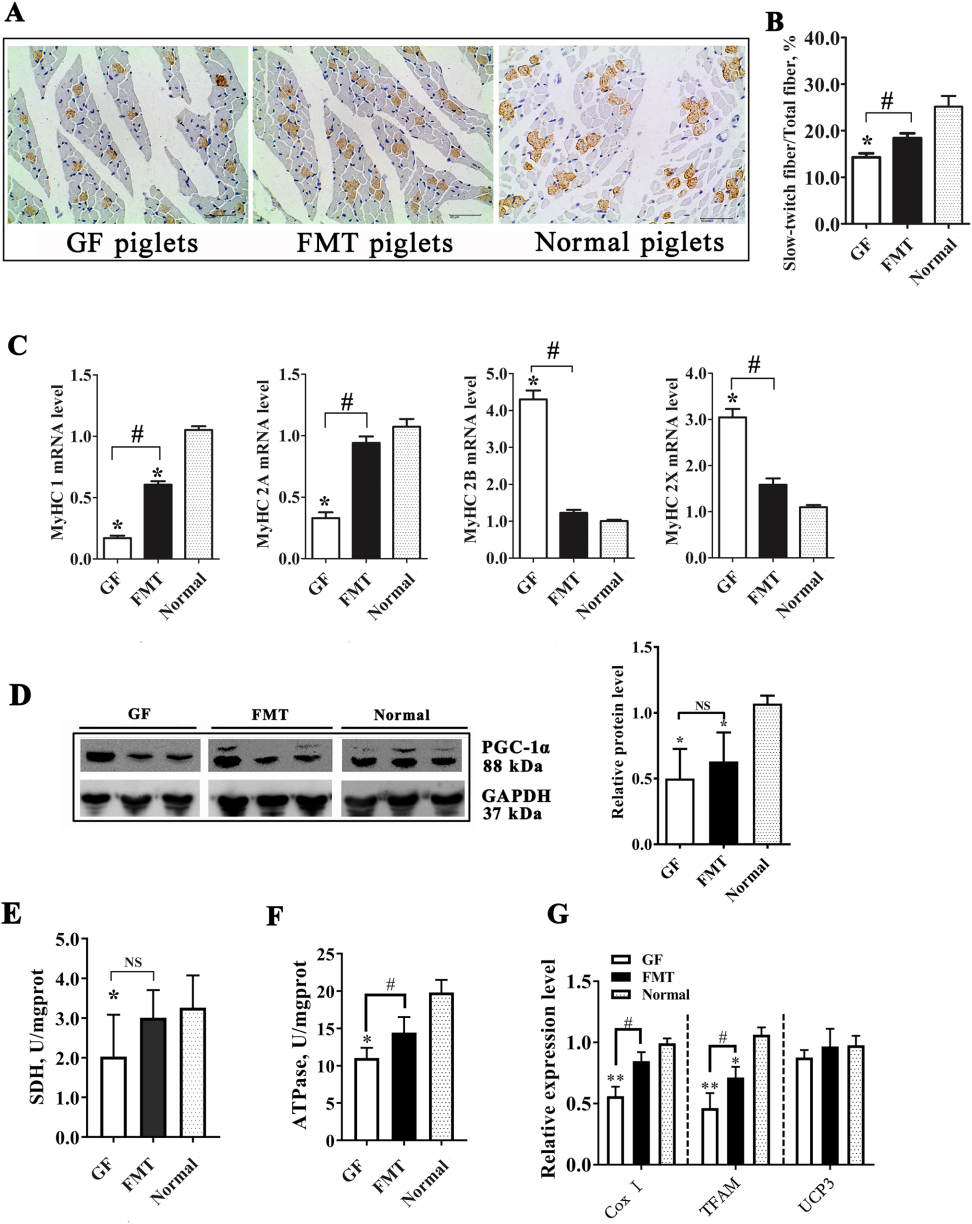
Change in the muscle fiber type in GF piglets. (**A**) Immunohistochemical analysis of muscle samples. The brown spots indicate slow-twitch muscle fibers. (**B**) Proportion of slow-twitch muscle fibers in the total muscle fibers. The full-length blots are presented in Supplementary Fig. 2. (**C**) mRNA levels of different MyHC genes in the muscle tissues of piglets were detected by qRT-PCR. (**D**) Protein level of PGC-1α and relative quantitative analysis of the protein level. (**E**) Enzyme activity of succinate dehydrogenase (SDH) in muscle. (**F**) Enzyme activity of Na^+^K^+^-ATPase in muscle. (**G**) Changes in the expression of the cytochrome c oxidase I (Cox I), transcription factor A mitochondrial (TFAM), and uncoupling protein 3 (UCP3) genes in muscle tissue. N = 3, the data are presented as the means ± s.e.m. *P < 0.05, **P < 0.01 compared to the normal piglets (control). ^#^*P* < 0.05 between the GF piglets and FMT piglets. *NS* not significant, *GF piglets* germ-free piglets, *FMT piglets* GF piglets with fecal microbiota transplantation.

Mammalian skeletal muscle fibers can be divided into two main categories: slow-twitch (type 1) and fast-twitch (type 2) fibers. These fibers have different structures and metabolic characteristics, and the fast-twitch muscle fibers can be further categorized into types 2a, 2b, and 2 × based on the MyHC gene type^41–43^. We observed a decrease in the proportion of slow-twitch muscle fibers in the GF piglets compared with the control piglets and a partial restoration of slow-twitch muscle fibers in the FMT piglets (*P* < 0.05, Fig. 7A,B). qRT-PCR analysis indicated a downregulation of the MyHC1 and 2a genes and an upregulation of the MyHC 2b and 2 × genes in the muscles of GF piglets relative to those of normal piglets (Fig. 7C). These results suggested that deleting gut bacteria caused a change in the composition proportions of muscle fiber types in animals.

Peroxisome proliferator-activated receptor-γ coactivator 1α (PGC-1a), a mitochondrial biogenesis marker and transcriptional coactivator, could regulate genes that are involved in energy metabolism in different tissues. PGC-1a also serves as a major control factor causing the slow twitching of fibers in muscles^44,45^. PGC-1a was downregulated at different levels in the muscles of GF piglets and FMT piglets compared to normal piglets (*P* < 0.05, Fig. 7D). Moreover, we also observed reductions in the activities of the key mitochondrial enzymes succinate dehydrogenase (SDH) and Na^+^K^+^-ATPase in the muscle tissue of GF piglets (*P* < 0.05, Fig. 7E,F). In addition, the expression levels of several mitochondrial function genes (cytochrome c oxidase I, Cox I; transcription factor A mitochondrial, TFAM) in the muscle tissue of GF piglets were also reduced by gut microbiota deletion (Fig. 7G). These results overall suggested that the percentage change in muscle fiber type caused by the absence of intestinal microbiota was partly due to the decrease in oxidative metabolic capacity and the reduction in energy utilization in muscle tissue. Additionally, the significant decrease in the SCFA content in the GF piglets (Fig. 2F) could contribute to the changes in muscle fiber, especially the reduction in butyric acid, which is a regulator of fiber type^46^.

## Discussion

In recent years, intestinal microbes have received unprecedented attention because of the increasing number of reports indicating that the gut microbiota can potentially affect host gut health. An imbalance in the intestinal microbiota and the invasion of harmful bacteria will thus cause diseases in humans and animals. Dozens of diseases, such as obesity^47^, diabetes^48^, Parkinson's disease^49^ and depression^50^, have been closely associated with intestinal bacteria. Thus, an increasing number of studies have focused on the interference of gut microbiota in the development of diseases or in altering the physiological state of the host^51,52^. Our present study demonstrated that the intestinal microbiota contributed to the healthy growth and development of young pigs and that GF piglet were hypogenetic. Similar to other previous studies based on GF animal models^15,53^, we also found that the immune system of GF piglets was impacted, with several immune organs developing dysplasia.

As an important functional organ in humans and animals, skeletal muscle maintains body temperature, facilitates locomotion, stabilizes posture, provides energy storage and expenditure, and communicates with other parts of the body through the release of growth factors and cytokines^54,55^. Additionally, muscle tissues of livestock are an important meat resource for humans. Today, we know that microbiota reside in the gastrointestinal tract of animals and are inextricably linked to the growth and function of muscle tissue. It has been speculated that gut bacteria are able to affect the growth and function of host muscle tissues in two major ways: (1) changing the secretion of hormones related to the muscle (e.g., insulin and IGF1) by motivating the gut-brain axis and/or (2) producing functional metabolic compounds (e.g., SCFAs) that act as signaling factors for muscle cells. Given that gut microbes have been shown to control development and metabolism in muscle, promoting and maintaining the muscle growth of livestock by interfering with the intestinal microbiota or using microbe metabolites is an important aspect of agricultural production that needs to be further considered^56^.

Some reports have indicated that several probiotics are beneficial for muscle growth and resistance to muscle inflammatory diseases. For example, supplementation with *Bacteroides thetaiotaomicron* increased muscle mass in humans under standard or high-fat intake conditions^57^. Long-term supplementation with *Lactobacillus plantarum* TWK10 in mice increases muscle mass, grip strength and endurance swimming time^58^. However, no direct quantitative analysis of the integral contributions to the gut microbiota on the growth of muscles in large animals has been conducted to date. This study first revealed the differences in muscle growth between GF pigs and pigs with gut microbes, thus reaffirming the importance of gut microbiota in the maintenance of muscle growth and development.

In rodents, some studies have shown the effects of gut bacteria on muscle growth and function by using GF mice or other gnotobiotic animals. Recently, Lahiri et al*.* reported that GF mice displayed reduced skeletal muscle weight compared to conventionalized mice that had a gut microbiota and transplanting the gut microbiota of conventionalized mice into GF mice restored their muscle mass^14^. Another study also found that the muscle weight of GF mice was less than that of specific pathogen-free (SPF) mice and *Bacteroides fragilis* (BF)-gnotobiotic mice. The exercise performance of GF mice significantly decreased compared with that of SPF and BF mice^59^. Similarly, a reduction in the muscle mass of GF piglets was observed in this study, and GF piglets had only half of the *longissimus dorsi* muscle mass of normal piglets of the same age with gut microbiota. The significantly increased activity of the CDK enzyme and upregulated Glut 4 gene implied abnormal changes in glucose and energy expenditure in the muscle tissue in the GF piglets. FMT helped GF pigs obtain a gut microbiota, which may remedy their dysplasia to some extent.

Bäckhed observed that the absence of the gut microbiota in mice increased fatty acid catabolism in muscle tissue, which may be caused by AMPK activation^60^. In a study by Lahiri, GF mice exhibited atrophy of skeletal muscles, decreased expression of insulin-like growth factor 1, and reduced expression of genes associated with skeletal muscle growth and mitochondrial function^14^. We observed a similar decrease in mitochondrial function in GF piglets, which was proven by the reductions in SDH activity and transcription of mitochondrial genes. Moreover, the high-throughput sequencing analysis identified 1156 altered genes in GF piglets that were associated with the regulation of muscle growth and development, fatty acid oxidation, cell cycle, metabolism, G-protein signaling, and immune function. Importantly, twenty-nine DEGs were closely enriched in the pathways for muscle growth and development. In particular, remarkable increases in KLF15 and GATM promoted muscle atrophy in GF pigs. Other changed genes were involved in the control of autophagy and ubiquitin-mediated protein degradation, such as Atrogin1 and Murf-1. Furthermore, a reduction in IGF1 blood content and inactivation of the IGF1/AKT/mTOR pathway also provide the driving force in the process of muscle growth restriction and muscle atrophy in GF piglets. These results help us to understand the mechanism by which intestinal microbiota affect the growth and function of muscle tissue. However, due to the shortage of qualified samples, we did not perform RNA-seq analysis on the muscle of the FMT piglets, which obscured our knowledge about microbial colonization changing the gene expression profiles in muscle to some extent.

Unexpectedly, we observed that the gut microbiota affected the muscle fiber type. The percentage of slow-twitch muscle fibers in the *longissimus dorsi* muscle of GF piglets was significantly less than that of normal piglets and FMT piglets. PGC-1α, a key factor in the control of muscle fiber type, was significantly reduced in GF piglets. These changes may be caused by the weakening of mitochondrial function and energy turnover in the muscle tissue of GF piglets. In a previous study, the gut microbiota of obese pigs was transplanted to GF mice, and the results showed that the mice had fewer fast-twitch muscle fibers but more slow-twitch muscle fibers than the control mice^61^. Supplementation with *Lactobacillus* also affects muscle fiber type and enhances the athletic stamina of mice^53^. In addition, the metabolites of gut microbes could also affect the proportion of differential fiber types in muscle tissue. Supplementing butyrate in the diet of mice caused an increase in slow-twitch fibers and the upregulation of the MyHC1 gene in the *gastrocnemius* muscle^62^. Supplementing β-hydroxy-β-methylbutyrate (HMB) (a leucine metabolite) increased the fast MyHC protein levels and decreased the slow MyHC protein in mice^63^. However, our data showed that the butyric acid content in GF piglets was only approximately one-tenth that of normal piglets, which may be one of the causes for the change in muscle fiber type. Therefore, we believe that the rich and stable gut microbiota is an important factor in maintaining the muscle fiber type of the host.

FMT is a simple and fast method for gut bacterial transplantation, and FMT has been widely used to treat diarrhea caused by *Clostridium difficile* infections in clinical medicine for years^64^. However, some studies based on animal models have broadened the application of FMT in recent years. Different reports have shown that FMT effectively transfers the donor’s gut microbiota to the recipient and then changes the health and physiological state of the recipient. The application of FMT from obese or lean humans to GF animals induced changes in body composition that were consistent with the phenotype of their donor, highlighting the transmissible effects of metabolic phenotype via microbial exchange^65^. Moreover, several reports have shown the effectiveness and convenience of FMT in pigs^66–69^. These studies have confirmed that FMT could be used to promote pig growth and treat diarrhea, especially in little swine. Our recent work has also proven that transplanting intestinal microbiota from healthy adult pigs accelerated the body weight gain and metabolic maturity of piglets^70^. We thus hypothesize that it is very possible to shape the intestinal microbiota of pigs by gut bacterial transplantation to improve their meat production performance and pork quality, which is undoubtedly extremely beneficial to human needs. We also must strengthen the assessment of the safety and repeatability of FMT in future studies because inadequate or incorrect FMT will inevitably lead to the invasion of harmful bacteria and development of disease in the recipient^71,72^.

In addition, many limitations were observed in this work and some interfering factors were not well controlled in the present study. The differences in nutritional intake, acquired immunity and living environment between GF and normal piglets would also partially affect muscle growth and metabolism. The effect of colonization and changes in the intestinal microbiota on muscle will be explored in our subsequent study based on a more standard and rigorous experimental system.

## Conclusions

In conclusion, we show that the removal of the gut microbiota leads to muscle loss and muscle atrophy and changes the composition percentage of muscle fiber type in piglets. The reasons for the changes are very complex and possibly involve multidirectional physiological changes, including nutrient absorption and metabolism, hormone secretion, and energy expenditure. The colonization of gut microbes via FMT partly restores the growth and function of muscle tissue in GF piglets to some extent. We believe that an abundant and stable intestinal microbiota contributes to the growth, development and function of muscle tissues in humans and animals.

## Materials and methods

### Ethics statement

The study was approved by Ethics Committee of the Chongqing Academy of Animal Science (No. 20170035), all methods were carried out in accordance with relevant guidelines and regulations. This study was carried out in compliance with the ARRIVE guidelines.

### Animals

Six newborn GF piglets were obtained via hysterectomy from a multiparous Chinese Bama sow (a common local Chinese small pig breed). Three of them were exposed to microorganisms through the FMT method (FMT piglets). GF piglets and FMT piglets were reared in positive-pressure sterile fiberglass isolators (Class Biologically Clean Ltd., WI, USA; three piglets per isolator) with a heated floor at 32–35 °C. They were fed to satiety 5–7 times a day with an autoclave-sterilized cow’s milk-based formula prepared from condensed milk (Co60 γ-irradiated sterile). Three other normal piglets that were born and lived in a conventional agricultural environment were used as controls for the natural breastfed piglets. The main nutrient levels were similar in the formula milk (for GF and FMT piglets) and breast milk. Limited by the size of sterile isolators, our current study could observe only the situation of piglets at their early growth stage. All GF, FMT and normal piglets were euthanized under isoflurane anesthesia at 25 days of age. Their venous blood, *longissimus dorsi* muscle, and colon contents were collected and cryopreserved for subsequent analyses. The body weight and length of all piglets were recorded weekly and on the day of sacrifice.

### FMT

Eight candidate adult donor pigs were used in the current study and consumed a regular diet without antibiotics and probiotics for 6 weeks prior to faeces collection. Hog cholera virus, porcine parvovirus, porcine circovirus-2, porcine reproductive syndrome virus, respiratory syndrome virus, pseudorabies virus, foot and mouth disease virus, and mycoplasma hyopneumoniae were detected in the pigs. Finally, one pig without any pathogen was used as the trial donor for FMT.

FMT was performed five times on three GF infant pigs (from day 3 to day 7). Briefly, 2 g of fresh stool was collected from the healthy donor pig and then homogenized in 50 mL of cold saline water, filtered and then settled by gravity for 5 min, and 1 mL of supernatant (mean count 8 × 10^8^ CFU/mL) was administered via gavage to each recipient piglet. Parts of the faecal samples were stored at − 80 °C for microbiome DNA extraction.

### 16S rRNA gene sequencing

Illumina sequencing of the 16S rRNA gene was performed to characterize the microbial diversity and community composition. Sequencing was performed by Majorbio Bioinformatics Technology Co., Ltd., Shanghai, China. Total genomic DNA from the fecal suspension, feces sample of the donor pig and colon contents of the recipient piglets (FMT pigs) were extracted for PCR amplification using specific primers with the barcode (16S V3–V4). The PCR resulting amplicons were sequenced on the Illumina MiSeq platform. The sequences were clustered into OTUs (Operational Taxonomic Units) with 97% consistency, and a representative sequence of OTUs was selected. Sequences for each OTU were picked and aligned using QIIME version 1.8 and GreenGenes version 13_8 as the reference database. The microbial diversity and composition were analysis as previously described^23^.

### qRT-PCR assay

To determine the expression levels of protein coding genes in the muscle tissues, total RNA was extracted from fresh muscle tissues by using an RNAplus kit (Takara, Dalian, China). Total RNA was reverse-transcribed to cDNA using a PrimeScript RT Reagent Kit (Takara) according to the manufacturer's instructions. qPCR was performed using the Q6 qPCR system with SYBR Premix Ex Taq II (Takara) and normalized using the GAPDH gene as the endogenous control. All reactions were set to 3 replicates, and the expression levels of genes were expressed as fold-change using the 2^−△△CT^ method. Primer information is listed in Supplementary Table 1.

### Western blotting

Protein abundances of some key regulatory factors involved in the growth and development of muscle were determined by the standard western blot method with the GAPDH protein as a loading control. The anti-MyoD (#13812), anti-INSR(#74118) and anti-GAPDH (#2118) primary antibodies and horseradish peroxidase-conjugated secondary antibodies (anti-rabbit IgG, #5127; anti-Mouse IgG, # 58820) were obtained from CST (Cell Signaling, MA, USA). The anti-MYF5 (# bs-6936R) and anti-PGC 1α (# bs-1832R) primary antibodies were obtained from Bioss (Bioss Biotech, Beijing, China). The anti-myogenin (MyoG, # ab1835) primary antibody was obtained from Abcam Biotech (Abcam Biotech, Cambridge, UK). The anti-IGF1R (#20254-1-AP), anti-AKT (#10176-2-AP), anti-P-AKT (#66444-1-Ig), anti-mTOR (#66888-1-Ig), anti-P-mTOR (#67778-1-Ig) primary antibodies were obtained from Proteintech (Proteintech Biotech, Wuhan, China).

### Tissue sections and staining

As our previous description^73^, the muscle samples were embedded in optimal cutting temperature freezing medium (OCT, Sakura Finetek, CA, USA) at − 20 °C for 2 h, and then a cryostat (CM1950, Leica, Germany) was used to section the tissue at − 20 °C for histologic analysis. Hematoxylin–eosin (HE)-stained muscle sections (8 μm) were used to observe the morphology of muscle fiber. Images of the slides were captured by a digital microscope camera on a biological microscope (YS100, Nikon, Tokyo, Japan). An image analysis program (ImageProPlus 5.0, Media Cybernetics, MD, USA) was used to evaluate all parameters in the sections.

### Immunohistochemistry

The frozen sections of muscle samples were mounted on charged glass slides and processed for immunohistochemical detection of skeletal myosin (slow) using standard immunoperoxidase procedures. The mouse monoclonal anti-skeletal myosin (slow) antibody (#MAB1628) was purchased from Sigma-Aldrich (Sigma-Aldrich, MO, USA). The antibody was applied at 1:2000 dilution overnight at 4 °C. Diaminobenzidine (purchased from the Beyotime Institute of Biotechnology, China) was used as a chromogen to generate a brown precipitate attributable to its reaction with peroxidase. All slides were counterstained with hematoxylin, rinsed, dehydrated, mounted with Permount and then observed by light microscopy.

### RNA-sequencing analysis

High-throughput mRNA sequencing was used to identify the differences in the gene expression profiles in the muscles of the GF piglets and normal piglets. Total RNA from the *longissimus dorsi* muscle samples was isolated using a TRIzol reagent kit (Invitrogen, CA, USA) following the manufacturer’s instructions. RNA degradation and contamination were monitored using 1% agarose gels, and RNA purity was checked using a Nanodrop2000 spectrophotometer. A sequencing library was constructed from 3 μg of total RNA per sample. The libraries were generated using a Truseq RNA sample Prep Kit (Illumina, CA, USA) following the manufacturer’s instructions. All RNA libraries were sequenced on an Illumina HiSeq 4000 platform, generating 150-bp paired-end reads (Majorbio Bioinformatics Technology, Shanghai, China). After Illumina HiSeq sequencing, the per-sample clean reads were mapped back to the *Sus scrofa* reference genome. Gene expression was measured with HTSeq54 (v0.6.1) and normalized using the expected number of fragments per kilobase of transcript sequence per millions base pairs sequenced (FPKM) method. The DEGs were considered with *P adjust* ≤ 0.05 and an absolute value of log2 (fold-change) ≥ 1. The GO enrichment of the identified DEGs was analyzed by the GOseq R package, and GO terms with Q-values < 0.05 were considered significantly enriched. The protein network analysis of the DEGs was executed in the STRING protein function database (http://string-db.org/).

### Colonic SCFA contents analysis

The contents of SCFAs in the piglets’ colonic contents were determined by using an ISQ Lt GC–MS (Thermo Fisher, MA, USA) equipped with a flame ionization detector (FID). A TG WAX column (30 m × 0.25 mm × 0.25 um; Thermo Fisher) was used for separating the SCFAs. An injection volume of 5 μL of sample was automatically injected into the inlet, which was kept at 240 °C with a 75:1 split ratio. The carrier gas was helium at a flow rate of 1.0 mL/min. The FID and injector temperatures were 250 °C and 200 °C, respectively.

### Biochemical analysis

The concentrations of triglycerides, total cholesterol, low-density lipoprotein cholesterol (LDL-C), high-density lipoprotein cholesterol (HDL-C), and glucose in the piglets’ serum were detected by using a 7060 Automatic Analyzer (Hitachi, Tokyo, Japan). The concentrations of growth hormone, insulin, and IGF1 in the blood were evaluated by using commercial rat enzyme-linked immune sorbent assay (ELISA) kits (R & D Systems Co. Ltd., Shanghai, China).

### Enzyme activities of SDH and ATPase

The enzyme activities of Succinate dehydrogenase (SDH) and Na^+^K^+^-ATPase were estimated in muscle tissues using a Micro SDH Assay Kit and a Micro Na + K + -ATPase Assay Kit (Solarbio, Beijing, China) following the manufacturer’s instructions.

### Statistical analysis

All statistical analyses for data were performed using SPSS 22.0 software (IBM, NY, USA). Data are expressed as means ± s.e.m. Differences between groups were analyzed using a one-way ANOVA followed by Tukey–Kramer test. Comparisons of medians between non-normally distributed groups were performed using the Kruskal–Wallis H test. *P* < 0.05 was considered statistically significant.

## Supplementary Information

Supplementary Information.
