# Supplementary material for: The intestinal microbiota contributes to the growth and physiological state of muscle tissue in piglets
Source: Sci Rep. 2021 May 27;11:11237. doi: 10.1038/s41598-021-90881-5 (PMC8160342; doi:10.1038/s41598-021-90881-5)
Supplement: Supplementary file 1 — Supplementary Information. [file 41598_2021_90881_MOESM1_ESM.pdf]

The intestinal microbiota contributes to the growth and physiological state of muscle tissue in piglets

Renli Qi[1,2,3,#,*], Jing Sun[1, #], Xiaoyu Qiu[2], Yong Zhang[4], Jing Wang[1], Qi wang[1], Jinxiu Huang[1,2], Liangpeng Ge[1,2,3,*] & Zuohua Liu[1,2,*]

[1] Chongqing Academy of Animal Science, Rongchang, Chongqing, 40240, China

[2] Key Laboratory of Pig Industry Sciences, Ministry of Agriculture, Rongchang, Chongqing, 402460, China

[3] Chongqing Key Laboratory of Pig Industry Sciences, Rongchang, Chongqing, 402460, China

[4] Southwest University of Science and Technology, MianYang, Sichuan, 621010, China

* Corresponding Author, qirenli1982@163.com (R.Q); geliangpeng1982@163.com (L.G.); liuzuohua66@163.com (L.Z.)

Supplementary table 1. Sequences of qPCR primers

Gene	Sequence 5` → 3`
GPCR40	R: GCCGTGCTCTGTCCCATCTTG<br>F: ACACTCAGGGCAGCCAGGAAG
GPCR41	R: CGCTGCTCAACTTCCTCGTCTG<br>F: GTCTCCACTCGGGGCTTTTGC
GPCR43	R: GCTGCTGCTGCTGCCCTTG<br>F: TGCTGATGCCCGCCAGGAG
Glut4	R: CAACCAGCCTATGCCACCATCG<br>F: GCCCGTTCCACCAGCAACAC
CAT1	R: CATCAAAAACTGGCAGCTCA<br>F: TGGTAGCGATGCAGTCAAAG
CD36	R: TCGTGCCTATCCTCTGGCTT<br>F: TTCTACCAGGCCAAGGAGGT
COX1	R: ATTATCCTGACGCATACACAGCA<br>F: GCAGATACTTCTCGTTTTGATGC
TFAM	R: GCTTATAGGGCAGACTGGCAG<br>F: GACCATCCTTAGCTTCCTGAAA
MyHC1	R: AAGGGCTTGAACGAGGAGTAGA<br>F: TTATTCTGCTTCCTCCAAAGGG
MyHC2A	R: GCTGAGCGAGCTGAAATCC<br>F: ACTGAGACACCAGAGCTTCT
MyHC2B	R: ATGAAGAGGAACCACATTA<br>F: TTATTGCCTCAGTAGCTTG
MyHC2X	R: AGAAGATCAACTGAGTGAACT<br>F: AGAGCTGAGAAACTAACGTG
GAPDH	R: GAAGGTCGGAGTGAACGGAT<br>F: CATGGGTAGAATCATACTGGAACA

Supplementary Figure 1: The full-length blots of Figure 3.

A. INSR

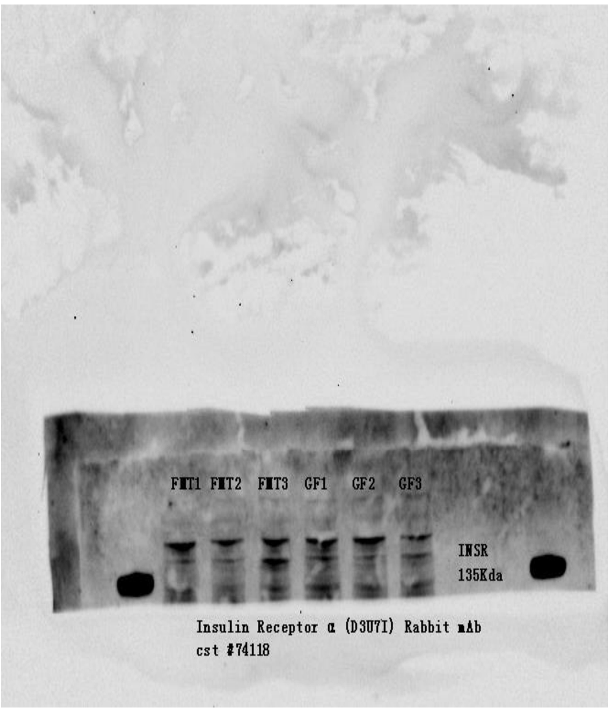

(GF Pigs and FMT pigs)

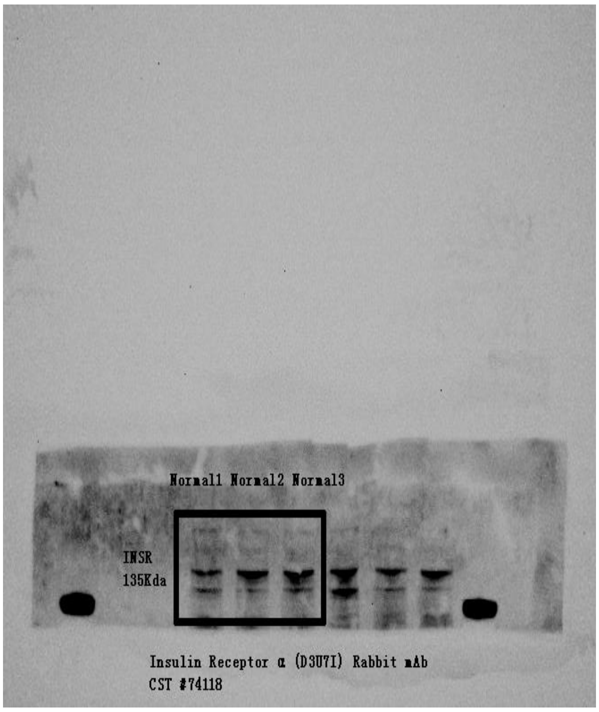

(Normal pigs)

B. MyoD

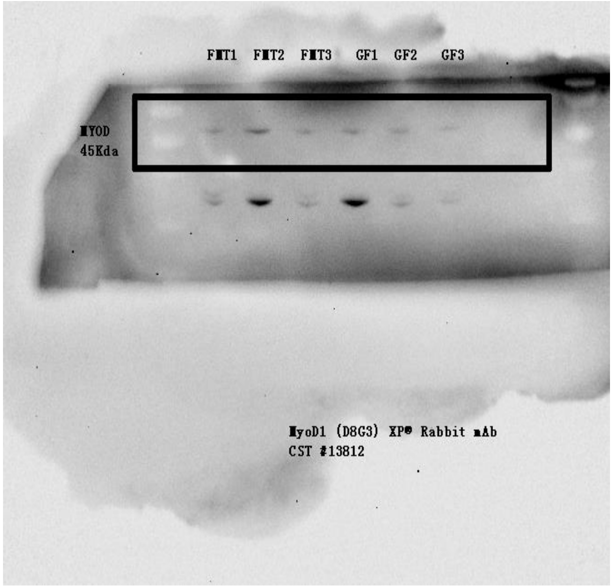

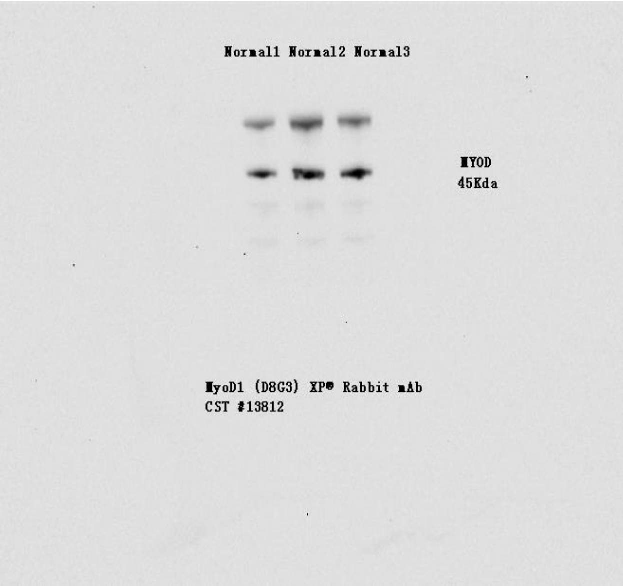

C. MYF5

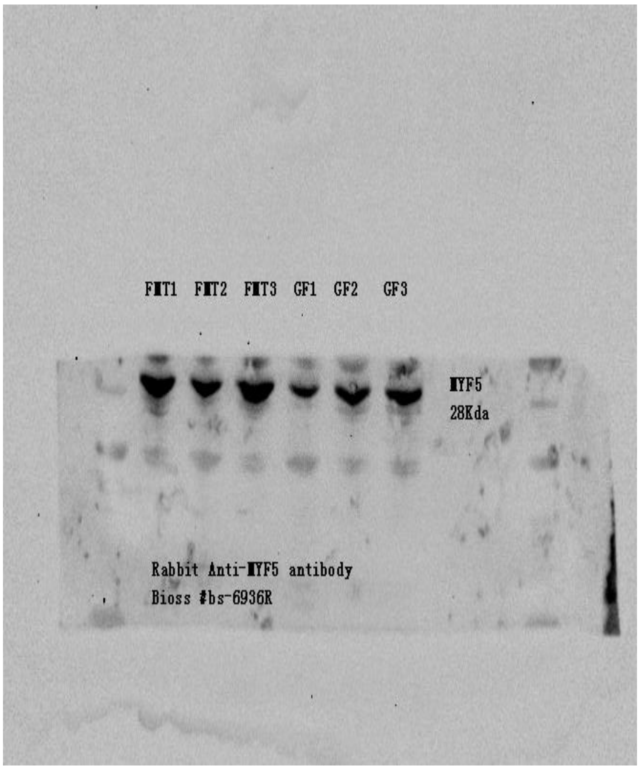

(GF Pigs and FMT pigs)

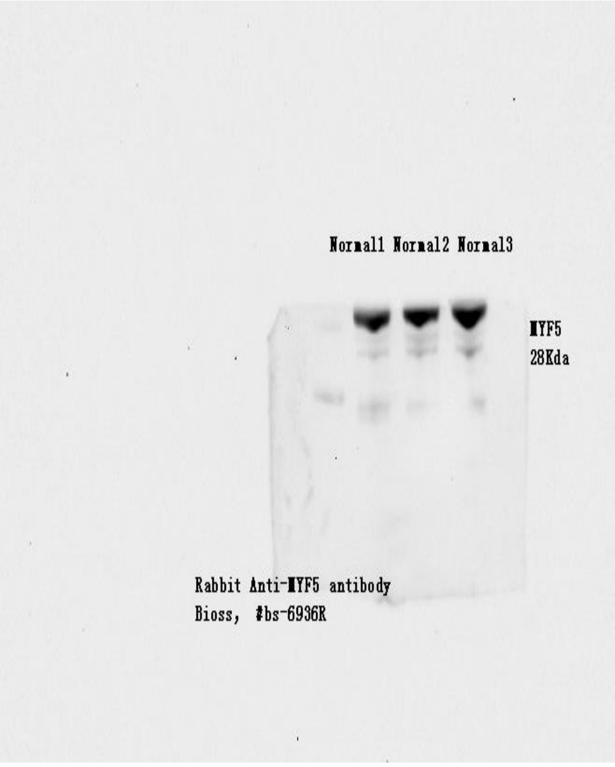

(Normal pigs)

D. Myogenin

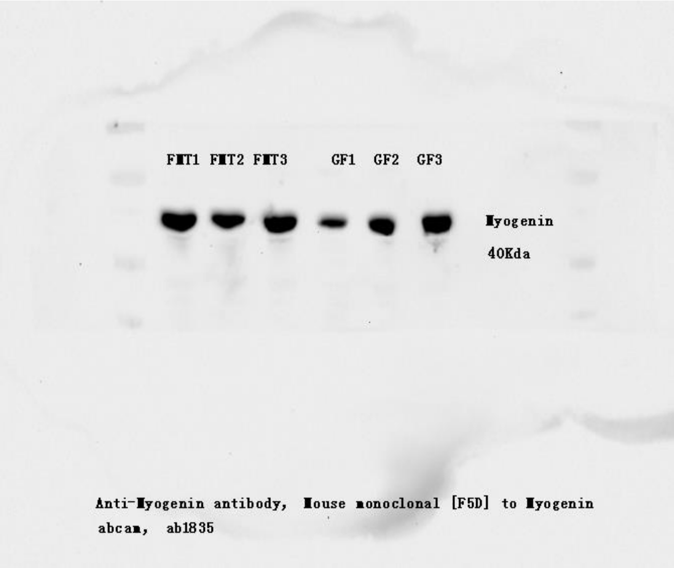

(GF Pigs and FMT pigs)

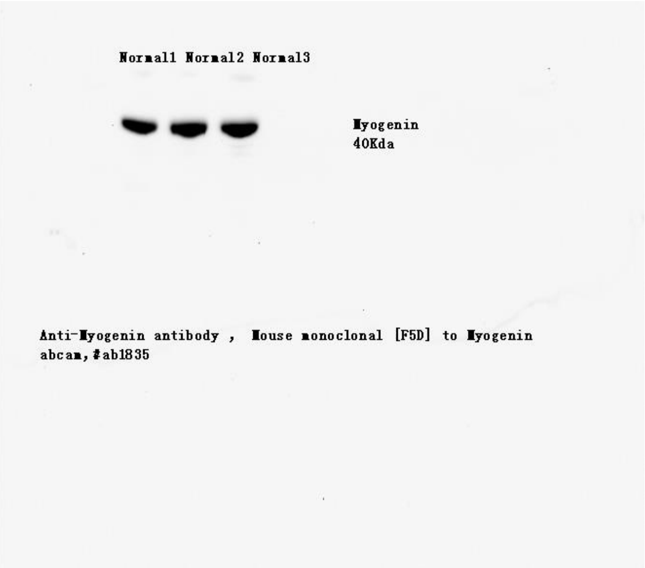

(Normal pigs)

E. GAPDH

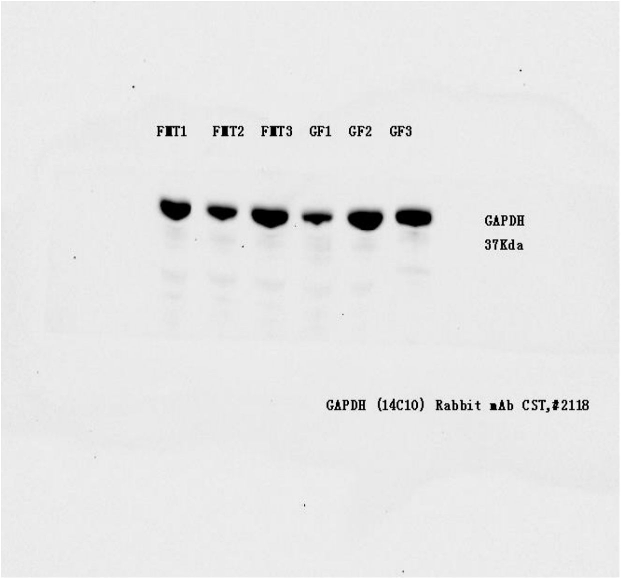

(GF Pigs and FMT pigs)

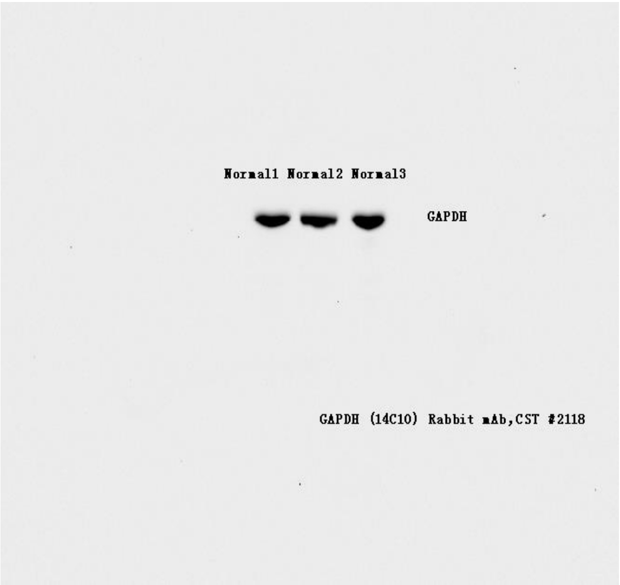

(Normal pigs)

Supplementary Figure 2: The full-length blots of Figure 6.

IGF1R

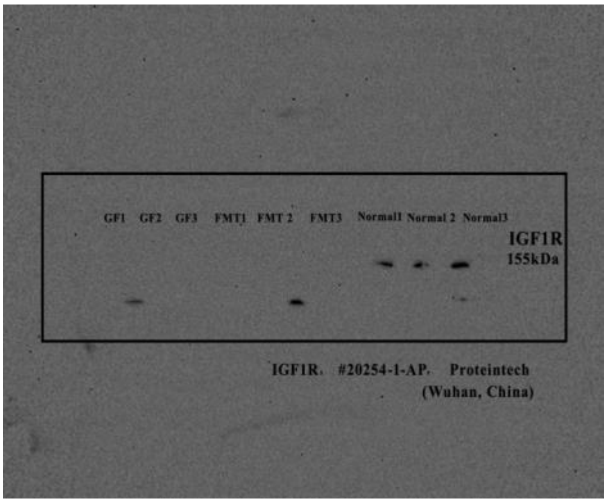

P-AKT

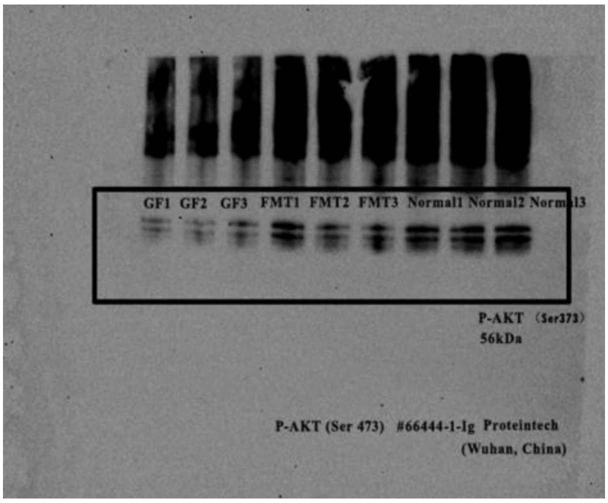

AKT

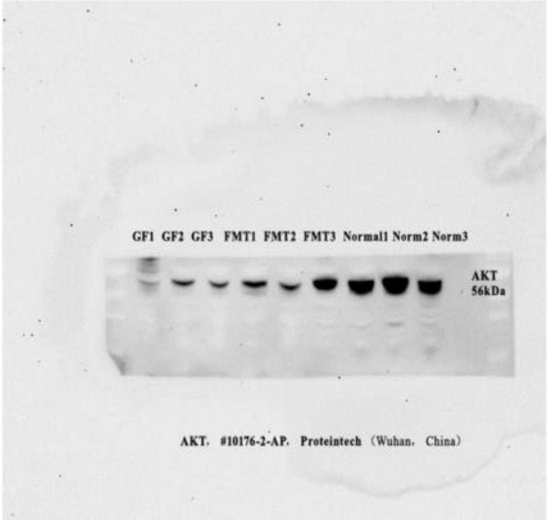

P-mTOR

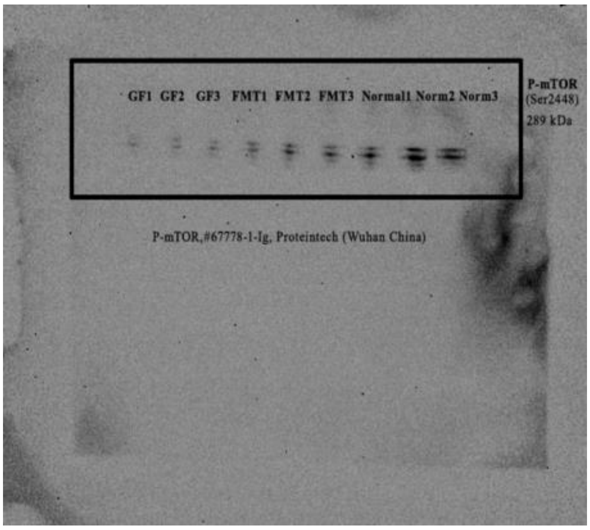

mTOR

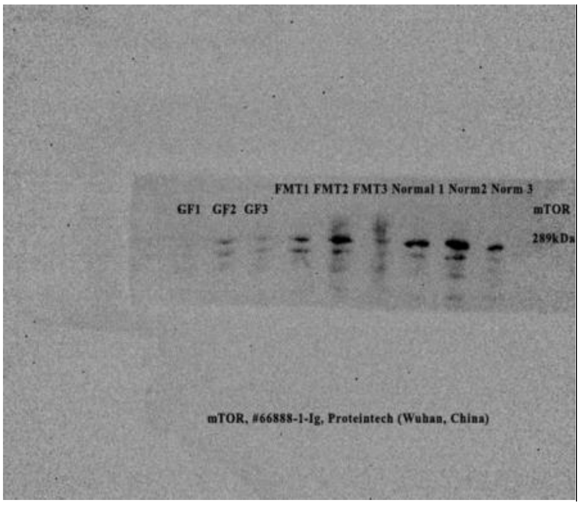
GF1 GF2 GF3
FMT1 FMT2 FMT3 Normal 1 Norm2 Norm 3
mTOR
289kDa
mTOR, #66888-1-Ig, Proteintech (Wuhan, China)

GAPDH

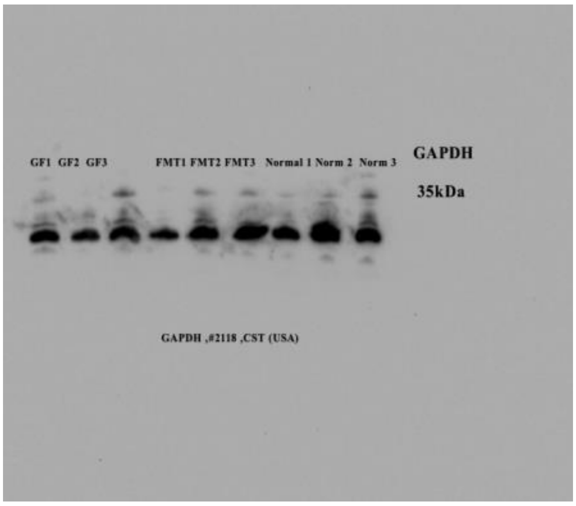
GF1 GF2 GF3
FMT1 FMT2 FMT3
Normal 1 Norm 2 Norm 3
GAPDH
35kDa
GAPDH ,#2118 ,CST (USA)

Supplementary Figure 3: The full-length blots of Figure 7.

A. PGC-1a

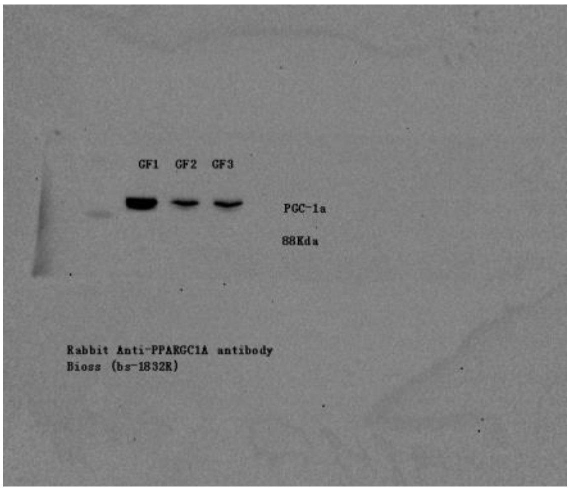

(GF Pigs)

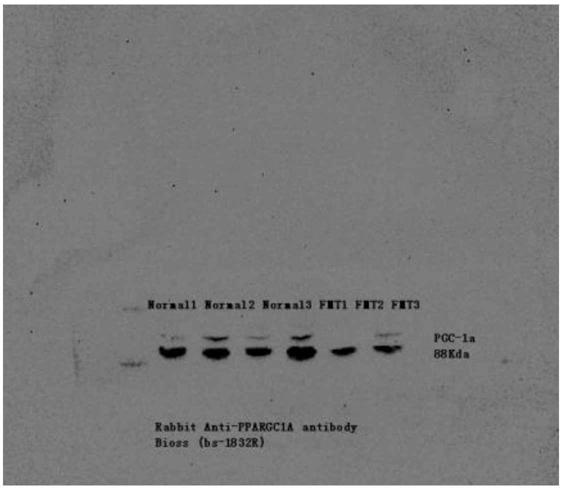

(Normal pigs and FMT pigs)

B. GAPDH

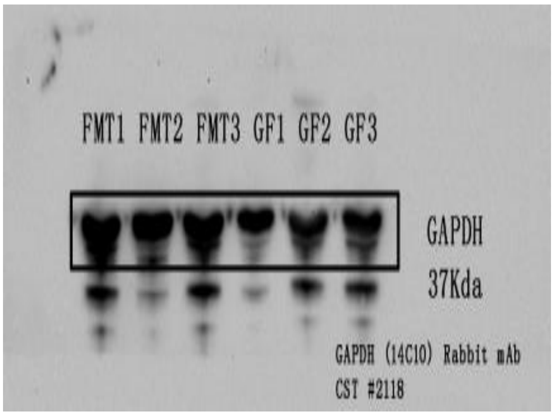

(GF Pigs and FMT pigs)

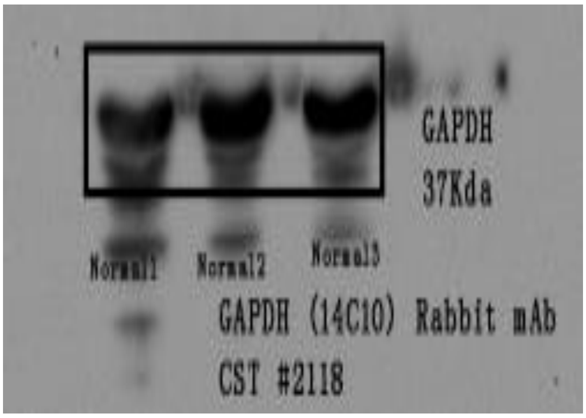

(Normal pigs)
